# Impact of periodontal treatment on the RANKL/OPG ratio in crevicular fluid

**DOI:** 10.1371/journal.pone.0227757

**Published:** 2020-01-27

**Authors:** Andrés López Roldán, José Luis García Giménez, Francisco Alpiste Illueca

**Affiliations:** 1 Dept. of Periodontics, Faculty of Medicine and Dentistry, University of Valencia, Valencia, Spain; 2 Center for Biomedical Network Research on Rare Diseases (CIBERER), CIBER-ISCIII, Madrid, Spain; 3 Dept. of Physiology, Faculty of Medicine and Dentistry, University of Valencia, Valencia, Spain; 4 INCLIVA Health Research Institute, Valencia, Spain; 5 Epigenetics Research Platform, CIBERER-UV, Valencia, Spain; New York Medical College, UNITED STATES

## Abstract

**Aim:**

Alveolar resorption is one of the most important events in periodontitis. Osteoclast activity is regulated by the ratio between receptor activator of NF-*κ*B ligand (RANKL) and osteoprotegerin (OPG). The aim of this study was to evaluate changes in the RANKL/OPG ratio in crevicular fluid after periodontal treatment.

**Material and methods:**

A total of 15 patients with periodontitis were included in the study group. Samples were collected from an area with active periodontitis and a healthy area. The RANKL and OPG levels were measured before and after periodontal scaling and root planing (SRP) treatment. The study group was compared to the control group, which included 10 patients without periodontitis. ID Clinicaltrial.gov: NCT03787875.

**Results:**

A decrease in the RANKL level was found in areas with active periodontitis after periodontal treatment, but no change in the OPG level was observed.

Therefore, the treatment induced a decrease in the RANKL/OPG ratio in sites with destructive periodontal activity.

**Conclusions:**

Periodontal treatment acts on the RANKL/OPG ratio by decreasing osteoclastogenesis. These results encourage the use of these molecules for periodontal diagnosis, monitoring and treatment.

**ID Clinicaltrial.gov:**

NCT03787875.

## Introduction

Alveolar bone resorption is one of the main mechanisms underlying the pathogenesis of periodontitis [1,2]. Alveolar resorption is caused by an increase in and a preponderance of osteoclast activity. At the molecular level, osteoclast activation is regulated by the interplay of three molecules that constitute the RANK/RANKL/OPG axis [3–5]. The receptor activator of nuclear factor-κB (RANK) receptor is a transmembrane protein expressed in both mature osteoclasts and their progenitors, and its binding to its ligand (RANKL) determines osteoclast differentiation and activation [6]. RANKL is a protein of the tumour necrosis factor family [7] that activates the RANK receptor. The osteoprotegerin ligand (OPG-L) is a soluble protein that functions as a decoy receptor for RANKL [6]; thus, OPG-L is an inhibitor of osteoclast formation [8].

These molecular markers have been studied in periodontitis. Previous studies showed higher RANKL levels in patients with periodontitis than in healthy subjects at both the immunohistological [9–16] and crevicular fluid (CVF) [13,17–23] levels. In contrast, lower OPG levels were found in subjects with periodontitis than in healthy subjects [10,11,13,14,17,18].

These results indicate that the RANKL/OPG ratio is higher at sites with periodontal activity.

However, little information is available concerning the effects of periodontal scaling and root planing (SRP) treatment on the levels of these molecular markers at the CVF level [24–26].

Therefore, the aim of the present study was to investigate variation in the RANKL and OPG levels in the CVF after periodontal scaling and root planning treatment.

## Material and methods

### Study design

A case-control study was proposed. A group of periodontally healthy subjects was compared to a group of patients with periodontitis before and after scaling and root planing.

Two sites were studied in each subject with periodontitis: one affected site (with periodontitis) and one healthy site (without periodontal disease); the latter site served as a control. Thus, the study units were the healthy and pathological sites before and after SRP treatment.

The masking was triple blind, including the researcher in charge of sample collection, the laboratory technician and the statistician.

Due to the absence of pre-established reference values for the RANKL and OPG concentrations in periodontal health, the results obtained were compared to a control group of subjects with periodontally healthy conditions during the second part of the study.

This protocol was approved by the ethics committee of the University of Valencia (Spain) according to the Declaration of Helsinki on April 2014. Written informed consent was obtained from the study subjects. The recruitment of patients was conducted between November 2015 and January 2016. The study began in February 2016 and was followed up until November 2016. The authors confirm that all ongoing and related trials for this intervention are registered (ID Clinicaltrial.gov: NCT03787875).

### Sample selection

The sample size was determined in relation to the main variable (the RANKL and OPG concentrations); currently, no reference values are available for the concentrations of these parameters under healthy or periodontal disease conditions. Therefore, to determine the sample size, we used the results of previously published studies. Bostanci et al. (2011) and Buduneli et al. (2009) (sample sizes of 27 and 20 subjects, respectively) analysed the concentration changes of these parameters in the CVF before and after basic periodontal treatment[24,25].

Therefore, 25 subjects were included in the final sample in our study. The subjects were recruited consecutively at the Periodontics Unit at the Faculty of Medicine and Odontology of the University of Valencia. The age range for the selection of the patients was between 45–70 years, subjects of caucasian race and sex parity was sought.

The control group included 10 periodontally healthy subjects without signs or symptoms of periodontal disease, such as attachment loss, probing depth greater than 3 mm, bleeding on probing, in any teeth except third molars. As well as no radiographic evidence of bone loss.

The study group included 15 subjects diagnosed with mild or advanced chronic periodontitis [27]. Each periodontal patient had one non-affected single-rooted tooth (healthy site) and another single-rooted tooth with periodontitis (periodontitis site) with the following features:

Healthy site: a single-rooted tooth with probing depths below or equal to 3 mm without recession and without bleeding on probing.
Periodontitis site: another single-rooted tooth from the same patient with probing depth of 5–6 mm or greater and clinical attachment loss equal to or greater than 6 mm and bleeding upon probing.

The exclusion criteria were as follows: subjects with aggressive periodontitis; systemic diseases or consumption of drugs affecting bone metabolism (osteoporosis, arthritis, hormonal treatment, bisphosphonates, anti-inflammatory drugs, immunosuppressants … etc); antibiotic, anti-inflammatory or contraceptive treatment for the last three months prior to study initiation; antiplatelet therapy for the last 7 days prior to study onset; primary or secondary occlusal trauma; periapical or periodontal abscess in some of the teeth included in the study; any type of periodontal treatment within the last 6 months; under orthodontic treatment; smoking; and pregnant or breastfeeding.

### Periodontal clinical examination

The examination was performed by only one explorer using a Williams type manual periodontal probe (PQ-OW 208 396, Hu-Friedy^®^, USA). The probing depth, recession and attachment loss were measured for six sites per tooth. The “Gingival Bleeding Index” of the six sites per tooth was used to assess bleeding upon probing [28]. The Silness and Loe index [28] was used to assess dental plaques.

A complete radiographic series (18 radiographs) was performed for the study group with the ORing paralleling system (Dentsply^®^) and digital phosphor plates (Durr dental ^®^). Two bitewing horizontal radiographs were taken in the control group.

### Periodontal treatment

In the study group, periodontal scaling and root planning treatment of the 4 quadrants was performed for two consecutive weeks without the use of antiseptics or antibiotics, and the patients were instructed in oral hygiene, including both brushing techniques and proximal hygiene. Four weeks after the last SRP, the patients were examined clinically, and samples of crevicular fluid were collected again.

### Study sequence

Day 0- Subjects were chosen based on the inclusion and exclusion criteria. Crevicular fluid samples were collected.

Day 7- CVF samples were collected from the selected sites in both groups, and the samples were processed.

Day 14- Periodontal treatment (study group): scaling and root planing of 2 quadrants and provision of oral hygiene instructions.

Day 21- Periodontal treatment (study group): scaling and root planing of the 2 remaining quadrants and provision of oral hygiene instructions.

Day 49- Control group: clinical record and crevicular fluid sample collection and processing.

### Collection of crevicular fluid

The Periotron^®^ 8000 was calibrated prior to sample collection [29].

In the study group, 4 CVF samples were collected, 2 for each single-rooted tooth with the clinical conditions described above. Two samples from one single-rooted tooth were collected from the control group.

Supragingival plaque was removed with a sterile curette, sluiced, isolated with cotton rolls and dried with air to avoid contamination with saliva. The tip of the Periopaper^®^ was inserted into the sulcus or periodontal pocket until resistance was found and maintained for 30 seconds; tips dyed with blood were discarded. Subsequently, the CVF volume was measured by the Periotron 8000^®^.

In the following step, each sample was allocated into a sterilised Eppendorf tube with 100 microlitres of buffer containing phosphate-buffered saline (PBS) with protease inhibitors (C.N. P8340 Sigma, MO, USA). The Eppendorf tubes were centrifuged at 15,000 rpm for 5 min; then, another 100 microlitres of buffer was added, and the tubes were centrifuged for 5 min at 15,000 rpm. Finally, 200 microlitres of each sample was stored at –80°C prior to processing.

### Sample processing

RANKL and OPG were quantified using an enzyme-linked immunosorbent assay (ELISA). The technical specifications of the RANKL (total Human ELISA, BioVendor) and OPG (Bender MedSystems) kits were followed. The total RANKL and OPG concentrations were assessed.

### Statistical analysis

A Brunner-Langer model for longitudinal data was estimated for the study group, with the time and site of the mouth assuming the role of intra-subject factors. The main effects of these factors and the interaction between the two factors were evaluated with an ANOVA-type statistic (ATS) test of the model.

The Mann-Whitney test was used to compare the distribution of a parameter between the control group and the study group in an area of the mouth and at a particular time point.

The significance level used in the analysis was 5% (α = 0.05). The statistical test of the Brunner-Langer model reached a power of 0.44 with the aim of detecting a mean magnitude difference that was significant (compatible with the effect size f = 0.25) assuming a 95% confidence level. This difference is regarded as the comparison of an intra-subject factor. For the Mann-Whitney test for the same conditions with a significance level of 5%, considering an effect size of 1 (mean), the obtained power to detect differences between the control and study groups was 0.66,5%.

## Results

### Clinical outcomes before (T1) and after (T2) periodontal treatment

Table 1 shows the probing depth, clinical attachment level and bleeding upon probing prior to localisation of the study unit.

**Table 1:** Clinical outcomes.

	PPD- T1 (mm)	PPD-T2 (mm)	CAL- T1 (mm)	CAL-T2 (mm)
C	2.30 ± 0.48	-------	2.30 ± 0.48	-------
HL	2.53 ± 0.51	2.26 ± 0.70	3.40 ± 1.40	3.20 ± 1.52
PL	7.00 ± 1.25	3.93 ± 1.16 *	8.00 ± 1.72	5.00 ± 1.48*

* Significant differences at the pathologic location after periodontal treatment in the probing depth and clinical attachment loss.

The sample was distributed among 7 men and 8 women in the study group. In the control group were included 5 women and 5 men. The mean age was 59.5 ± 5.5 in the study group and 49.3 ± 3.7 in the control group.

The mean probing depth of the location with periodontitis in the study group decreased significantly after treatment (p<0.001); a trend towards a decrease in the healthy location was observed, although the difference was not significant (p = 0.046). Despite the decrease in the probing depth at the pathologic location after treatment, the depth did not reach the level of the healthy location after treatment; this difference was significant (p<0.001).

When we compared the study group with the control group, we found that the mean probing depth of the healthy location of the study group did not differ from that of the control group either before (p<0.338) or after treatment (p<0.978).

At the pathologic location of the study group, although the decrease in the probing depth from T1 to T2 was significant, the T2 values were not equal to those found for the control group; therefore, the differences were significant (p<0.001).

The gain in clinical attachment at the pathologic location of the study group increased significantly after treatment (p<0.001). A trend towards a gain was observed in the healthy location but was not significant (p = 0.083). After treatment, the attachment gain was higher at the pathologic location than at the healthy location (p = 0.004), but the pathologic location value did not reach the value of the healthy location after treatment (p<0.001).

The healthy location (p = 0.026) and the pathologic location (p<0.001) showed differences in the clinical attachment levels before periodontal treatment compared to the control group. After periodontal treatment, only the healthy location showed values similar to those found for the control group (p = 0.026).

Initially, bleeding occurred upon probing in all pathologic locations of the patients from the study group (100%), whereas bleeding only occurred in 6.7% of the locations after SRP treatment (p<0.001).

The clinical outcome considering the subject as the study unit showed that the mean probing depth in the study group was 4.08 ± 0.48 mm before periodontal treatment and was 2.98 ± 0.43 mm after periodontal treatment, which represented a significant difference (p<0.001).

The mean probing depth value in the control group was 2.51 ± 0.21 mm, which differed significantly from the depths in the study group before (p<0.001) and after (p = 0.005) treatment.

Regarding the percentage of bleeding, no bleeding was found in the control group. The study group the full mouth bleeding score showed values of 47.73% before treatment (T1) and 6.6% after basic periodontal treatment (T2). A significant decrease was observed in the percentage of bleeding between T1 and T2 in the study group (p<0.001).

Although the bleeding decreased significantly with treatment, it remained higher at T2 than the values in the periodontally healthy subjects (control group) (p = 0.002).

The mean plaque index in the control group was 0 because it was one of the inclusion criteria. The study group plaque index showed values of 2,67 ± 0,49 in T1 and 0,33 ± 0,48 after periodontal treatment (T2). A significant decrease was observed in the plaque index between T1 and T2 in the study group (p<0.001).

The mean volume of crevicular fluid in the control group was 0.10 ± 0.04 μl. The volume in the study group before treatment (T1) was 0.16 ± 0.1 μl in the healthy location and 0.74 ± 0.31 μl in the pathologic location. After basic periodontal treatment (T2), the values were 0.15 ± 0.01 μl in the healthy location and 0.35 ± 0.19 μl in the pathologic location. A significant decrease was observed in the volume between T1 to T2 in the pathologic location (p <0.001).

### Changes in RANKL (Table 2 and Fig 1)

**Fig 1:**
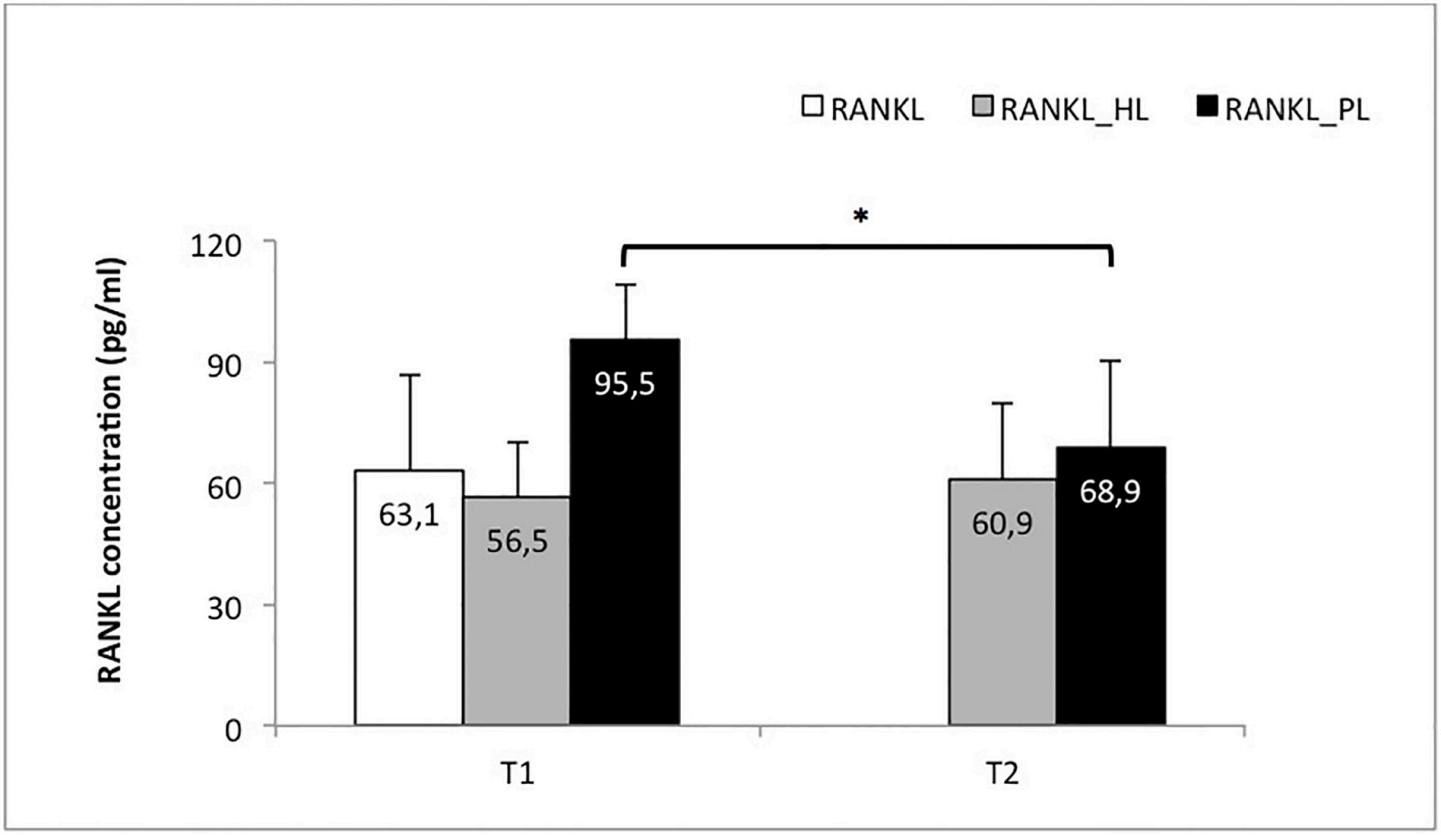
RANKL outcomes. Fig 1 Mean concentration of RANKL (pg/ml) in the control group (RANKL) and the study group before (T1) and after the basic periodontal treatment (T2) at the healthy location (RANKL_HL) and the pathologic location of the subjects with periodontitis (RANKL_PL). * Significant differences in the RANKL_PL group after performing the basic periodontal treatment.

**Table 2:** RANKL outcomes.

	Pre-treatment (T1) (pg/ml)	Post-treatment (T2) (pg/ml)
RANKL	63.1 ± 23.8	------------------------------------
RANKL_HL	56.5 ± 13.4	60.6 ± 18.8
RANKL_PL	95.5 ± 13.8	68.9 ± 21.1*

* Significant differences in the RANKL_PL group after performing the basic periodontal treatment.

Significant differences in the RANKL concentration were found in the study group (p<0.001) between the healthy location (RANKL_HL) and the pathologic location (RANKL_PL) before the periodontal treatment (T1), with the highest values found for the location with the periodontal affectation.

After the periodontal treatment, the RANKL concentration of the subjects from the study group was maintained almost unchanged from T1 (pretreatment) to T2 (post-treatment) at the healthy location (RANKL_HL) (p = 0.233); however, the RANKL concentration decreased significantly at the pathologic location (RANKL_PL). Thus, the RANKL values significantly decreased from T1 to T2 at the pathologic location (p = 0.005).

In addition, when analysing T2, we found no difference in the RANKL concentration between both locations (RANKL_HL and RANKL_PL) at the post-treatment time point (p = 0.173). Therefore, the basic periodontal treatment in the pathologic locations led to a decrease in the RANKL level until values equal to the healthy locations were obtained (pre- or post-treatment).

When the control group (RANKL) was compared to the study group, we found that the soluble ligand RANKL levels were significantly higher at the pathologic locations in the subjects with periodontitis before undergoing the treatment (p<0.001). When comparing the control group (RANKL) with the healthy location of the study group (RANKL_HL), a p value of 0.461 was obtained; therefore, no significant difference was found.

In conclusion, a significant decrease in the RANKL concentration at the pathologic locations in the periodontal patients was found after periodontal treatment, although no significant differences were observed in the healthy locations of these patients (p = 0.367). We conclude from these data that the RANKL levels in the healthy subjects (RANKL) do not differ from the RANKL levels in the subjects with periodontitis after periodontal treatment regardless of the location [healthy (RANKL_HL) or with periodontitis (RANKL_PL)].

### Changes in OPG (Table 3 and Fig 2)

**Fig 2:**
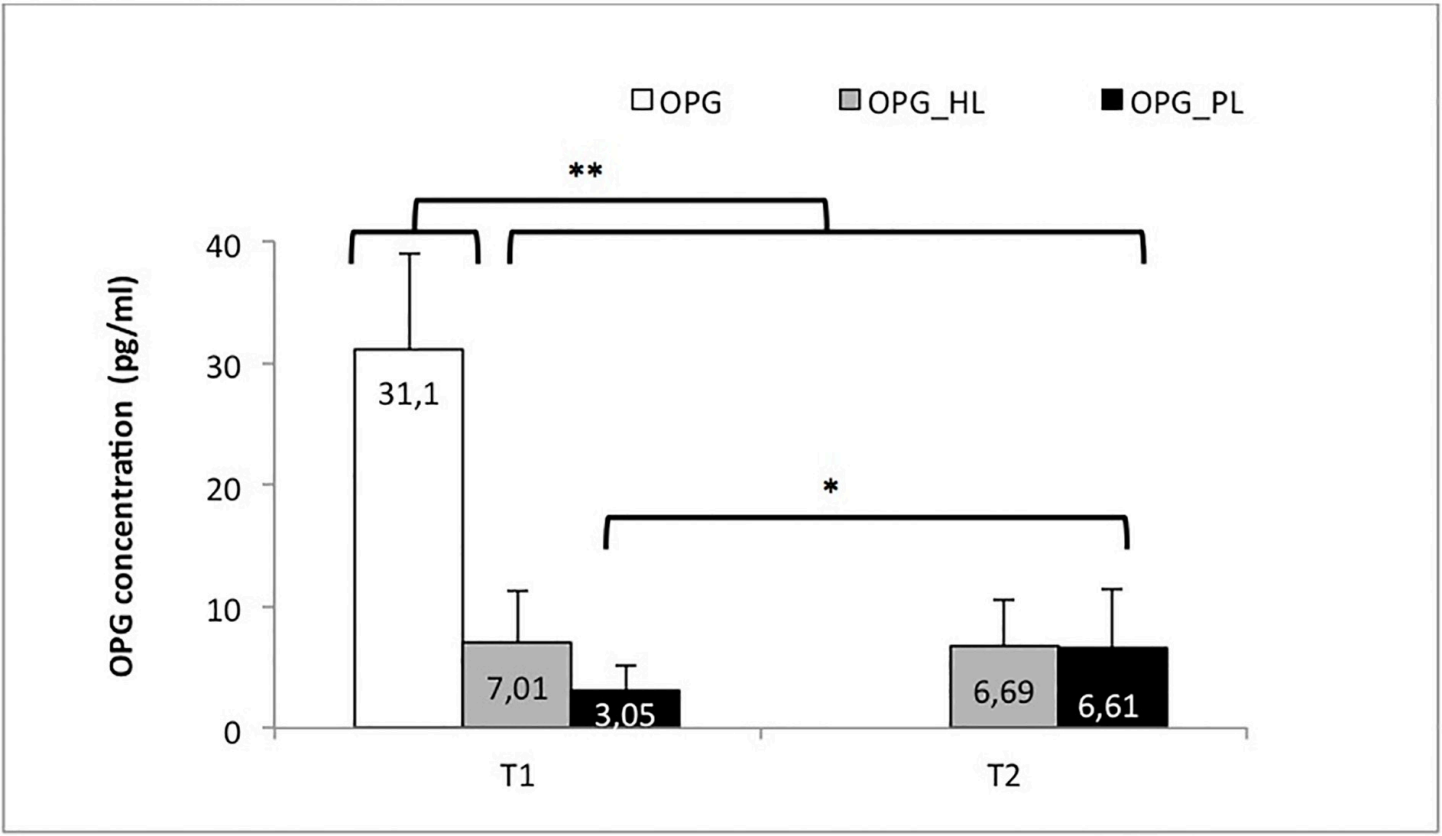
OPG outcomes. Fig 2. Mean OPG (pg/ml) concentration according to group and time point: control group (OPG) and study group before (T1) and after basic periodontal treatment (T2). As function of the group: the healthy location in the subjects with periodontitis (OPG_ZS) and the pathologic locations in the subjects with periodontitis (OPG_ZE). * Significant differences in the OPG_ZE group between T1 and T2. ** Significant differences between the control group and the other groups.

**Table 3:** OPG outcomes.

	Pre-treatment (T1) (pg/ml)	Post-treatment (T2) (pg/ml)
OPG	31.10 ± 7.89	------------------------------------
OPG_HL	7.01 ± 4.33	6.69 ± 3.89
OPG_PL	3.05 ± 2.08	6.60 ± 4.76*

* Significant differences in the OPG_PL group after performing the basic periodontal treatment.

Prior to periodontal treatment (T1), significant differences (p<0.001) were found in the study group between the healthy locations (OPG_HL) and the locations with periodontitis (OPG_PL), with lower OPG levels in the OPG_PL.

After basic periodontal treatment (T2), an increase was found in the OPG levels at the periodontitis locations in the study group, and this change was significant (p = 0.009). In contrast, no significant changes in the OPG levels were found at the healthy locations.

Thus, we found homogeneity of the post-treatment OPG levels between the pathologic and healthy locations (p = 0.55).

The OPG levels were higher in the control group than in the study group regardless of whether the subjects had undergone periodontal treatment.

### Changes in the RANKL/OPG ratio (Table 4 and Fig 3)

**Fig 3:**
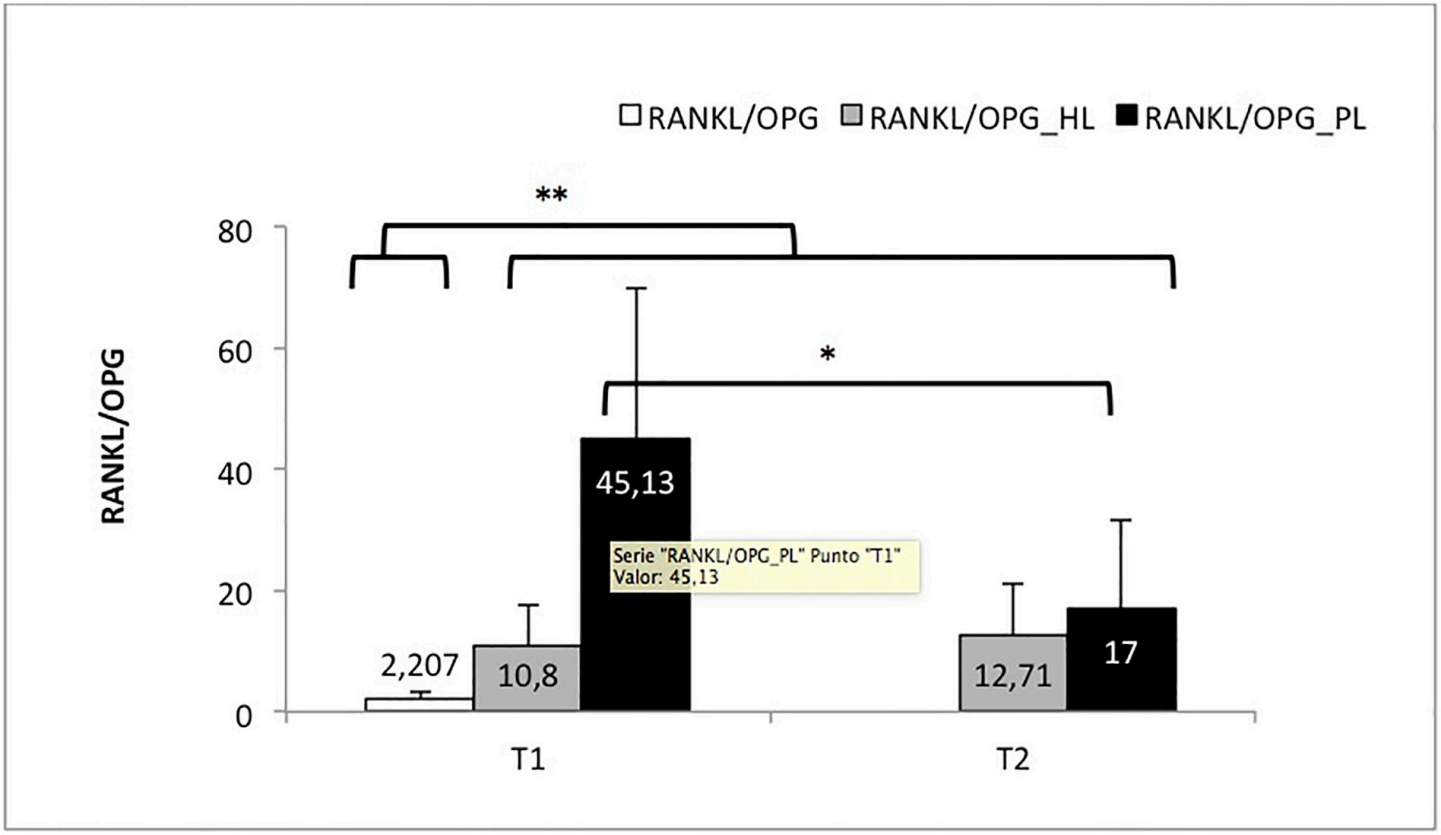
RANKL/OPG outcomes. Fig 3. Evolution of the RANKL/OPG ratio according to the group and time point: control group (RANKL/OPG) and study group before (T1) and after (T2) basic periodontal treatment. As a function of the group: healthy location of subjects with periodontitis (RANKL/OPG_HL) and pathologic location of the subjects with periodontitis (RANKL/OPG_PL). * Significant differences in the RANKL/OPG_PL group between T1 and T2. **Significant differences between the control group and the other groups.

**Table 4:** RANKL/OPG outcomes.

	Pre-treatment (T1)	Post-treatment (T2)
RANKL/OPG	2.20 ± 1.13	------------------------------------
RANKL/OPG_HL	10.8 ± 6.79	12.71 ± 8.43
RANKL/OPG_PL	45.13 ± 24.74	17 ± 1 4.15*

* Significant differences in the RANKL/OPG_PL group after basic periodontal treatment.

Significant differences were found in the RANKL/OPG ratio between the healthy and pathologic locations of the study group before periodontal treatment (p<0.001), with higher ratios observed in the pathologic locations.

A significant decrease in the ratio (p = 0.005) was found from pretreatment (T1) to post-treatment (T2) at the pathologic locations of the study group. However, no changes were found in the ratio at the healthy locations (p = 0.570).

In the group of study patients, no differences were found between the healthy and pathologic locations after SRP treatment (p = 0.307).

Finally, when comparing the control group and study group, we found that the RANKL/OPG ratio distribution of the control subjects was always significantly lower than the ratio of any other comparison group.

## Discussion

According to our study, the OPG values were higher in the healthy patients than in the treated or untreated patients in the study group. This result suggests that local inflammation may have a low influence on the OPG levels of the patients in the study group, as the values of the different samples were similar. These values show that subjects suffering from periodontitis have lower OPG levels than healthy patients; that is, subjects susceptible to periodontitis have lower basal OPG levels. The hypothesis that antiosteoclastogenic levels are higher in the control group is consistent with the results of the different estudies [18,26,30]. However, one factor that should be taken into account is that the subjects in the control group of our study were younger than the subjects in the study group; thus, age may be a factor that influences the basal OPG level.

Regarding the RANKL levels, higher values were found in the untreated location with the periodontal condition in the study group than in the other locations in both the study and control groups. In addition, except for the untreated affected locations, similar RANKL levels were found at all locations, with no significant differences among them.

This finding confirms that the RANKL levels are higher in locations with destructive periodontal activity, which results in higher osteoclast activity. These data are consistent with the results of previous studies [22,31,32].

However, when this local inflammation is absent, such as in the control group, at the healthy locations in the study group before treatment and at both locations (healthy and pathologic) post-treatment, the RANKL levels are lower and similar. This result confirms the significance of T cells as RANKL producers [12,15,33,34].

In conclusion, the RANKL/OPG levels are low in the control group, in the treated healthy locations and in the treated pathologic locations of patients suffering from periodontitis. The latter two levels were very similar but were not as low as the levels observed in the control group, which had the lowest levels. Furthermore, the RANKL/OPG ratio was high at the locations with untreated periodontitis, which was mainly determined by the high RANKL values.

The study design, including the healthy subjects and several stages of periodontal disease in the group of subjects with periodontitis, allowed us to conduct all of these comparisons and to uncover more information about periodontal pathogenesis.

This study assesses changes of these biomarkers after basic periodontal treatment. We do not have predetermined values of RANKL or OPG that mark the limit between periodontitis and periodontal health. Therefore, larger sample size studies are necessary to get this information.

However, the age of the subjects should be controlled, as age could be a factor to consider in bone metabolism.

The outcomes obtained in the present study in conjunction with the available literature provide a thorough understanding of the etiopathogenesis of periodontitis and indicate directions for prospective studies of biomarkers and even potential therapeutic targets, which will allow more personalised treatment.

## Supporting information

S1 Protocol Protocol: Impact of the periodontal treatment in ratio RANKL / OPG. (DOCX)

S2 Protocol Proyecto de tesis: Impacto del tratamiento periodontal en el ratio RANKL/OPG. (DOCX)

S1 STROBE checklist STROBE statement—Checklist of items that should be included in reports of observational studies. (DOC)
