# Supplementary material for: Impact of periodontal treatment on the RANKL/OPG ratio in crevicular fluid
Source: PLoS One. 2020 Jan 27;15(1):e0227757. doi: 10.1371/journal.pone.0227757 (PMC6984681; doi:10.1371/journal.pone.0227757)
Supplement: S1 Protocol — (DOCX) [file pone.0227757.s001.docx]

**PROTOCOL: IMPACT OF THE PERIODONTAL TREATMENT IN RATIO RANKL / OPG**

Author: Dr. Andrés López Roldán

Periodontics teaching unit. Department of Stomatology.

Faculty of Medicine and Dentistry. UNIVERSITY OF VALENCIA.

**INDEX:**

1. Introduction Pag. 3
2. Hypothesis Pag. 4
3. Objetives: Pag. 5
4. Material and method Pag. 6
5. Bibliography Pag.10

# INTRODUCTION

Periodontal diseases are a group of clinical diseases that affect the periodontal tissues of tooth, the two important clinical groups are gingivitis and periodontitis. Gingivitis is a localized inflammation of the marginal periodontium, however periodontitis is an inflammatory process that extends to the supporting tissues of the tooth and is characterized by apical migration of the junctional epithelium, the progressive destruction of the periodontal ligament and alveolar bone.

The importance of the study and treatment of this disease it is due to the high prevalence and clinical consequences; The continuous loss of periodontal support ends with tooth loss, but the effects of this pathology are not only limited to the odonto-stomatological field.

In the last decades, there have been great advances in the field of the etiopathogenesis of periodontal disease, being a complex entity where several factors intervene such as microbiology, hygienic habits, genetics or systemic modifying factors.

Basic research in periodontics follows a path in line with the advances that occur in other fields of science such as medicine, biology, biochemistry ... etc, so that in the periodontal discipline has passed in a few years of studies based in anatomopathology to today, where the study of pathology is increasingly addressed at the genetic and molecular level.

The main differentiating characteristic of periodontitis with respect to the rest of periodontal pathologies is the loss of periodontal support which explicitly involves the resorption of alveolar bone. Consequently, one of the main objectives of the treatment is to prevent the bone loss from.

Regarding the mechanism of bone resorption that occurs in periodontitis, it is known that several factors associated with the host immune response are implicated, as well as innumerable paracrine signals capable of stimulating or inhibiting bone loss.

But, undoubtedly, a great step in the knowledge of bone metabolism was the discovery, at the end of the 90s, of the RANK / RANKL / OPG axis, this being the cornerstone of the osteoclastogenesis process and, therefore, the regulator of bone resorption.

The RANKL (Activator Receptor for Nuclear Factor κ B Ligand **)** is a protein that stimulates the differentiation of osteoclasts that exerts its action by binding to the transmembrane receptor RANK (Activator Receptor for Nuclear Factor κ B), present in preosteoclasts, instead OPG (osteoprotegerin) acts as a decoy ligand binding to RANKL and inhibiting its osteoclastogenic action.

This discovery has been a great advance in the field of medicine, with applications in multiple diseases that occur with alterations in bone metabolism, including the oncological field.

In the specific field of periodontics, the first investigations arose at the beginning of the year 2000, the results of these studies have demonstrated the existence of high RANKL values and low levels of OPG locally in the affected sites of periodontitis.

Of all the methods used for the determination of these molecules, the studies with greater future clinical application are those that value the concentration of these molecules at the level of the crevicular fluid because it is a non-invasive technique.

At present, there are few publications that determine these mediators at the level of the crevicular fluid, in addition different methodologies have been used, which makes it difficult to obtain conclusions of clinical application. Although these publications have contributed important knowledge to better understand the mechanisms of regulation of bone remodeling of the periodontium, we have not found any study that assesses the impact of periodontal therapy on these markers. Therefore, the intention of this research work is to develop a clinical protocol for the taking of samples and determination of RANKL and OPG at the level of the crevicular fluid in patients with periodontitis, and later to determine the changes that occur in their concentrations when performs basic periodontal therapy.

Studies in this direction can provide new information of clinical interest that allows the use of RANKL and OPG as possible markers of the destructive activity of periodontitis, which would allow it to be used in diagnosis, prognosis and in the control of periodontal treatment.

# HYPOTHESIS

The importance of RANKL, RANK and OPG in the regulation of osteoclastogenesis is clear, which has allowed a better understanding of bone metabolism in both physiological and pathological conditions.

The study of possible determinants of periodontal destructive activity has been one of the major topics of interest in the field of periodontics. There are many studies that relate the levels of certain cytokines and other molecular mediators to destructive activity, but undoubtedly the discovery of the RANKL / RANK / OPG axis has allowed us to understand how the actions of various molecules involved in bone remodeling are integrated, because it is the final step that determines ostoclastic differentiation and activation.

The investigations published so far seem to indicate that RANKL concentrations are elevated in the periodontal tissues as well as in the crevicular fluid of patients with periodontitis. However, we have little information about whether the RANKL concentration can be related to the degree of severity of the pathology or to the phases of activity, or if there could be a different profile in the clinical forms of periodontitis, aggressive or chronic.

Regarding OPG concentrations, it seems clear that OPG levels are higher in periodontally healthy individuals or who only suffer from gingivitis. In patients with periodontitis, OPG levels are lower or even markedly lower in cases of aggressive periodontitis.

In view of the results of the investigations published up to now, it can be concluded that the RANKL / OPG ratio is higher in periodontitis than in gingivitis or in health situation. According to current knowledge, the cells responsible for RANKL expression at the periodontium level are fibroblasts (gingival and periodontal ligament), osteoclasts and osteoblasts; but in the presence of periodontitis, the main ones responsible for the expression of RANKL seem to be activated T and B lingotites. This fact would explain how the defensive system of the host against microorganisms of the bacterial biofilm would be the mediating mechanism of osteoclastogenesis (Osteoimmunology).

Despite the documented involvement of RANKL and OPG in periodontal disease, there are still several questions regarding the behavior of these mediators in the etiopathogenesis of this pathology, as well as the clinical utility they may have as markers of periodontal disease.

With respect to this last question, the response that occurs in these molecular mediators when performing basic periodontal therapy has not been studied.

Our main work hypothesis contemplates that basic periodontal treatment (scaling and root planing plus oral hygiene instructions) has an effect on the concentration of RANKL and OPG in the crevicular fluid of patients with periodontal disease, and there is a decrease in the RANKL ratio. OPG, reducing the absolute values of RANKL and increasing those of OPG.

# OBJECTIVES

The general objective of this research is to study the impact of basic periodontal treatment on the ratio of RANKL / OPG in the crevicular fluid of patients with periodontitis.

The main objectives are:

1º In the group of patients with periodontal disease we intend to study if the degree of periodontal health is related to the concentration of RANKL and OPG in the crevicular fluid. For this, we propose these specific objectives:

- 1. Assess the differences between healthy locations, with gingivitis and with periodontitis.
  2. Determine the differences in the levels of RANKL and OPG between locations with and without inflammation (bleeding on probing).
  3. To evaluate if the degree of severity of periodontitis (initial, moderate or advanced) is related to the levels of RANKL and OPG.
  4. To study the behavior of the levels of these molecular mediators after the basic periodontal treatment.

2º The second main objective is to study the differences in the levels of RANKL and OPG in the crevicular fluid between a group of periodontally healthy individuals and another group of patients with periodontal disease. For this we propose three specific objectives:

- 1. To compare the average levels of RANKL and OPG obtained in healthy individuals with those obtained in periodontal patients.
  2. To compare the levels of RANKL and OPG in healthy locations of healthy individuals with the levels obtained in healthy locations of periodontal patients.
  3. Contrast the levels of these molecular mediators obtained in periodontally healthy individuals with periodontal patients who have been treated with basic periodontal treatment.

# 5. MATERIAL Y METHOD

## SAMPLE

Sample of 30 subjets will be studied, which will be obtained from the staff of the Faculty and from the patients who come to first visits in the Periodontics Unit of the Odontology Clinic of the University of Valencia.

The 30 individuals will be divided into two study groups:

**Control sample** : 15 periodontally healthy individuals

**Study sample** : 15 patients with periodontal disease.

Inclusion criteria:

To be included in the study, individuals must read and sign the consent document, and they must commit to faithfully comply with the study protocol.

## 1.- Control sample:

Healthy individuals will be considered those who, both clinically and radiographically, do not present signs or symptoms of periodontal disease in any of their teeth.

## 2.- Sample study:

To be included in the sample, each patient must have been diagnosed with chronic periodontitis, and also have at least one uniradicular tooth for each of the following clinical situations:

- *Healthy tooth* : probing depths less than or equal to 3 mm, without recession and without bleeding on probing.
- *Tooth with gingivitis* : probing depths equal to or less than 3 mm, without recession and with bleeding on probing.
- *Teeth with moderate periodontitis* : Insertion loss greater than 3 mm. and less than 6 mm.
- *Tooth with severe periodontisis* : Insertion loss greater than 6 mm.

Exclusion criteria:

1.- Patients with aggressive periodontitis.

2.-Presence of systemic alterations or taking drugs that alter bone metabolism (osteoporosis, arthritis, hormonal treatment, bisphosphonates, anti-inflammatory drugs, immunosuppressants ... etc).

3.-Having received antibiotic, anti-inflammatory, contraceptive treatment in the last three months. 4.- Primary or secondary occlusal trauma in any of the teeth included in the studies.

5.- Have received periodontal treatment in the last 3 months. 6.- Be in orthodontic treatment.

Exclusion criteria during the study:

1.- Taking any drug that may alter the response to periodontal treatment (anti-inflammatories, antibiotics, antiseptic mouthwashes).

2.- Any drug that can alter bone metabolism.

In the same way, the impossibility on the part of the individual of strict compliance with the rules of the protocol will be an exclusion criterion.

## MATERIAL :

- Periodontal probe (Williams 10mm, Hu-Friedry ®)
- O-Ring radiographic parallelization system (Densply®)
- Intraoral radiography apparatus (E-wood®, Haigol-Dong, Korea)
- Perio paper strips ®: special paper strips to collect volumes of 0-1.2 ml crevicular fluid (Oraflow®, New York, USA)
- Periotron 8000®: electronic moisture micro-meter specially designed to assess the crevicular fluid, periodontal pocket fluid and salivary fluid (Oraflow®, New York, USA)
- Periotron professional: Software designed to pass the values given by Periotron 8000® to milliliters (Oraflow®, New York, USA)
- Δ 1.5 ml reaction vials (Ependorff®)
- Precision calibrated pipettes of 10-1000 µl and disposable tips (Ependorff®)
- Distilled or deionized water
- PBS (Phosphate buffer saline)
- Protease inhibitors (CN P8340 Sigma, Missouri, USA)
- Kit Elisa sRANKL and OPG (Biomedica Medizinprodukte®, Austria)
- Automatic plate washer
- ELISA reader with capacity to read absorbances from 450 nm to 620 nm.

## CLINICAL PARAMETERS:

- **Clinical history data:**

Patient's age Systemic pathologies Medication

Smoking habit

Hygienic habits (material, frequency, technique and time)

## Periodontal exploration:

Depth of probing: with a Williams millimetric manual periodontal probe, six points were recorded per tooth (disto-vestibular, mid-vestibular, mesio-vestibular, disto- palatal, mid-palatal, and mesio-palatal).

Recession: the distance from the amelocementary line to the gingival margin will be measured and recorded in the same points mentioned above (disto-vestibular, mid-vestibular, mesio-vestibular, disto-palatal, mid-palatal and mesio-palatal).

Clinical insertion level: Adding the aforementioned parameters we can find this parameter for each point.

Dichotomous index of bleeding on probing (Hemorrhage Index). Miller's mobility index.

Plaque index of Silness and Löe.

## Radiographic exploration:

In group cases, a complete radiographic series is made up of 18 radiographs of which 14 are periapical of incisors, canines, premolars and molars of all quadrants and 4 vertical bite fins of premolars and molars. All of them are performed with parallelized radiographic technique using the O-Ring parallelization system and intraoral X-ray apparatus.

In the control group, only two horizontal bite fins will be made to confirm the clinical diagnosis of absence of periodontal disease

## SAMPLING OF CREVICULAR AND PROCESSED FLUID:

On the same day of the clinical examination, 4 samples of crevicular fluid were collected from each of the uniradicular teeth with the 4 clinical situations described in the inclusion criteria.

Remove the supragingival plaque with a sterile curette without touching the gum, wash with plenty of water, insulate with cotton rollers and air dry to avoid contamination with saliva.

Insert the Perio Paper® tip until resistance is felt and leave it for 30 seconds. NOTE: If the paper is stained with blood or debris, the sample is contaminated and must be retaken.

Measure the volume of each sample using the Periotron 8000® and the appropriate software.

Place the paper tips in a sterilized Eppendorf tube, add 100 microliters of buffer (PBS together with protease inhibitors) to the sample and centrifuge at 15,000 for 5 min.

Add another 100 microliters of buffer and centrifuge again at 15,000 for 5 min.

Store 200 microliters of each sample at -80ºC until the ELISA test.

## ELISA TEST TESTS

Determination of the concentration of RANKL and OPG in the samples will be carried out through assay techniques by enzyme-linked immunosorbent assay ( In zyme-Linked ImmunoSorbent Assay ) ,

specifically, kits prepared from the Biomedica Medizinprodukte® commercial house will be used .

The analyzes will be carried out following the manufacturer's recommendations and the concentration will be determined once the quantification of RANKL and OPG has been carried out along with the volume found with the periotron.

## INTERVENTION IN THE SAMPLE CASES:

In the group of patients belonging to the sample cases, after having collected all clinical and radiological data and samples of crevicular fluid, basic periodontal treatment will be carried out. The treatment consists of scaling and root planing of the 4 quadrants without the use of antiseptics or local action antibiotics. As part of the treatment, patients will be instructed in oral hygiene, both in brushing techniques and in the use of interproximal hygiene measures. At 4 weeks they will be scheduled to re- evaluate clinically and take samples of the crevicular fluid again.

## SEQUENCE OF THE PROTOCOL:

Day 0 .- Confirmation of adequacy to the inclusion and exclusion criteria of the study. The patient will be explained what the study consists of, the protocol to be followed and the patient's consent. Realization of anamnesis, and collection of clinical and radiological data.

Day 7.- Samples of crevicular fluid will be taken from the selected areas and the samples will be processed.

Day 14.- Periodontal treatment (group of cases): 2-quadrant scaling and root planing and instructions on oral hygiene.

Day 21 .- Periodontal treatment (group cases): Scraping and root planing of the 2 remaining quadrants and oral hygiene instructions.

Day 49.- Group cases: Periodontal revaluation, clinical records and sampling of crevicular fluid. Processing of the samples

7. BIBLIOGRAPHY

Abu-Amer, Y., Erdmann, J., Alexopoulou, L., Kollias, G., Ross, FP, Teitelbaum, SL (2000) Tumor necrosis factor receptors types 1 and 2 differentially regulate osteoclastogenesis. Journal of Biological Chemistry.275 (35), 27307-10

Anderson, DM, Maraskovky, E., Billingsley WL, Dougall WC, Tometsko ME, Rous ER, et al. (1997) A homologue of the TNF receptor and its ligand enhance T-cell growth and dendritic-cell function. Nature 390, 175-179

Arron, JR, Choi, Y. (2000) Bone versus immune system. Nature 30; 408 (6812), 535-6

Armitage, GC (1999) Development of a classification system for periodontal diseases and conditions. Annals of Periodontolgy 4, (1): 1-6.

Bar-Shavit, Z. (2008). Taking a toll on the bones: Regulation of bone metabolism by innate immune regulators. Autoimmunity, 41 (3), 195-203.

Bostanci, N., Ilgenli, T., Emingil, G., Afacan, B., Han, B., Toz, H., Atilla, G., Hughes, FJ & Belibasakis, GN (2007a) Gingival crevicular fluid levels of RANKL & OPG in periodontal diseases: implications of their relative ratio. Journal of Clinical Periodontology 34, 370-376 .

Bostanci, N., Ilgenli, T., Emingil, G., Afacan, B., Han, B., To¨z, H., Berdeli, A., Atilla, G., McKay, I.,

Hughes, F . & Belibasakis, G. (2007b) Differential expression of RANKL

and OPG mRNA in periodontal diseases. Journal of Periodontal Research. 42, 287-293

Boyce, BF, Xing L. (2007) Biology of RANK, RANKL, and osteoprogesterin. Arthritis Research &

Therapy 9, 1-7

Brecx, MC, Fröhlicher, I., Gehr, P., Lang, NP. (1988) Stereological observations on

long-term experimental gingivitis in man. Journal of Clinical Periodontoly.15 (10), 621-7.

Choi, Y., Woo, KM, Ko, SH, et al. (2001) Osteoclastogenesis in enhanced by activated B cells but suppressed by activated CD8 + T cells. European Journal Immunology. 31, 2179-2188

Crotti, T., Smith, MD, Hirsch, R., Soukoulis, S., Weedon, H., Capone, M., Ahern, MJ & Haynes, D. (2003) Receptor activator NF kappaB ligand (RANKL) and osteoprotegerin (OPG) protein expression in periodontitis. Journal of Periodontal Research 38, 380-387.

Dougall, WC, Glaccum, M., Charrier, K., Rohrbach, K., Brasel, K., et al. (1999) RANK is essential for osteoclast and lymph node development. Genes and Development.13 (18), 2412-24

Ferrer J., Tovar, I., Martínez, P. (2002) Osteoprotegerin and RANKL / RANK System: the future of bone metabolism? Annals of Internal Medicine 19, 385-388.

Fuller, K., Murphy, C., Kirstein, B., Fox, SW, Chambers, TJ (2002) TNFalpha potently activates osteoclasts, through a direct action independent of and strongly

synergistic with RANKL. Endocrinology 143 (3), 1108-18

Garlet, GP, Cardoso, CR, Silva, TA, Ferreira, BR, Avila-Campos, MJ, Cunha, FQ, et al. (2006). Cytokine pattern determines the progression of experimental periodontal disease induced by actinobacillus actinomycetemcomitans through the modulation of MMPs, RANKL, and their physiological inhibitors. Oral Microbiology and Immunology, 21 (1), 12-20.

Glossary of Periodontal Terms. 4th Edition. The American Academy of Periodontology 2001

Grant, PR, & Mulvihill, JE (1972). The fine structure of gingivitis in the beagle. 3. plasma cell infiltration of the subepithelial connective tissue. Journal of Periodontal Research, 7 (2), 161-172.

Griffiths, G. (2004) Formation, accumulation and importance of gingival crevicular fluid. Periodontology 2000 6 32-41

Hasegawa, T., Yoshimura, Y., Kikuiri, T., Yawaka, Y., Takeyama, S., Matsumoto, A., et al. (2002).

Expression of receptor activator of NF-kappa B ligand and osteoprotegerin in culture of human periodontal ligament cells. Journal of Periodontal Research, 37 (6), 405-411.

Hodge, P., & Michalowicz, B. (2001). Genetic predisposition to periodontitis in children and young adults. Periodontology 2000, 26, 113-134.

Horton, JE, Raisz, LG, Simmons, HA, Oppenheim, JJ, & Mergenhagen, SE (1972). Bone resorbing activity in supernatant fluid from cultured human blood leukocytes. Science (New York, NY), 177 (51), 793-795.

Kanzaki, H., Chiba, M., Shimizu, Y., & Mitani, H. (2001). Dual regulation of osteoclast differentiation by periodontal ligament cells through RANKL stimulation and OPG inhibition. *Journal of Dental Research, 80* (3), 887-891.

Kawai, T., Matsuyama, T., Hosokawa, Y., Makihira, S., Seki, M., Karimbux, NY, et al. (2006). B and T lymphocytes are the primary sources of RANKL in the bone resorptive lesion of periodontal disease. The American Journal of Pathology, 169 (3), 987-998.

Khosla, S. (2001) Minireview: The OPG / RANKL / RANK system. Endocrinology 142, 5050-5055

Kinane, DF. Lindhe, J. (2005) Classification of periodontal diseases. In: Lindhe, J. Clinical periodontology and dental implantology. Ed. Panamericana, Buenos Aires. P. XII

Kinane, DF., Adonogianaki, E., Moughal, N., Winstanley, FP., Mooney, J., Thornhill, M. (1991). Immunocytochemical characterization of cellular infiltrate, related endothelial changes and determination of GCF acute-phase proteins during human experimental gingivitis. Journal of Periodontal Research. 26 (3), 286-8

Kitaura, H., Sands, MS, Aya, K., Zhou, P., Hirayama, T., Uthgenannt, B., Wei, S., Takeshita, S., Novack, DV, Silva, MJ, Abu-Amer , Y., Ross, FP, Teitelbaum, SL (2004) Marrow stromal cells and osteoclast precursors differentially contribute to TNF-alpha-induced osteoclastogenesis in vivo. Journal of Immunology.173 (8), 4838-46

Kong, YY, Boyle, WJ, Penninger, JM (2000). Osteoprotegerin ligand: a regulator of immune responses

and bone physiology. Immunology Today. 10, 495-502

Lam, J., Takeshita, S., Barker, JE, Kanagawa, O., Ross, FP, Teitelbaum, SL (2000) TNF-alpha induces osteoclastogenesis by direct stimulation of macrophages exposed to permissive levels of RANK ligand. Journal of Clinical Investigation. 106 (12), 1481-8

Lacey, DL, Timms, E., Tan, HL, Kelley, MJ, Dunstan, CR, Burgess, T., et al. (1998). Osteoprotegerin ligand is a cytokine that regulates osteoclast differentiation and activation. Cell, 93 (2), 165-176.

Lee, SH, Kim, TS, Choi, Y., & Lorenzo, J. (2008). Osteoimmunology: Cytokines and the skeletal system. BMB Reports, 41 (7), 495-510.

Lerner, UH, (2006) Inflammation-induced bone remodeling in periodontal disease and the influence of post-menopausal osteoporosis. Journal of Dental Research. 85 (7), 596-607.

Li, Y., Toraldo, G., Li, A., Yang, X., Zhang, H., Qian, WP, et al. (2007). B cells and T cells are critical for the preservation of bone homeostasis and attainment of peak bone mass in vivo. Blood, 109 (9), 3839- 3848.

Löe, H., Theilade, E., Jesen SB (1965). Experimental gingivitis in man. Journal of Periodontology 36, 177-187

Lorenzo, J., Horowitz, M., & Choi, Y. (2008). Osteoimmunology: Interactions of the bone and immune system. Endocrine Reviews, 29 (4), 403-440.

Lu, H.-K., Chen, Y.-L., Chang, H.-C., Li, C.-L. & Kuo, MY-P (2006) Identification of the osteoprotegerin

/ receptor activator of nuclear factor-kappa B ligand system in gingival crevicular fluid and tissue of patients with chronic periodontitis. Journal of Periodontal Research 41 : 354-360.

Martínez H, Jiménez F, Estrada C, Anaya J, Quiñones M. Cytokines and chemokines. In: Anaya J, Shoenfeld Y, Correa P. Autoimmunity and Autoimmune Disease. Ed. Corporation for biological research; 2005. p. 121-132

Michalowicz, BS, Diehl, SR, Gunsolley, JC, Sparks, BS, Brooks, CN, Koertge, TE, et al. (2000).

Evidence of a solid genetic basis for risk of adult periodontitis. Journal of Periodontology, 71 (11), 1699- 1707.

Mogi, M., Otogoto, J., Ota, N. & Togari, A. (2004) Differential expression of RANKL

and osteoprotegerin in gingival cervicular fluid of patients with periodontitis. Journal of Dental Research 83, 166-169.

Myers, DE, Collier, FM, Minkin, C., Wang, H., Holloway, WR, Malakellis, M., Nicholson, GC (1999) Expression of functional RANK on mature rat and human osteoclasts. FEBS Letters.463 (3), 295-300

Nagasawa, T., Kobayashi, H., Kiji, M., Aramaki, M., Mahanonda, R., Kojima, T., Murakami, Y., Saito, M., Morotome, Y., Ishikawa, I. ( 2002) LPS-stimulated human gingival fibroblasts inhibit the differentiation of monocytes into osteoclasts through the production of osteoprotegerin. Clinical and Experimental Immunology.130 (2), 338-44

Ogasawara, T., Yoshimine, Y., Kiyoshima, T., Kobayashi, I., Matsuo, K., Akamine, A., et al. (2004). In situ expression of RANKL, RANK, osteoprotegerin and cytokines in osteoclasts of rat periodontal tissue. Journal of Periodontal Research, 39 (1), 42-49.

Page, RC (1991) The role of inflammatory mediators in the pathogenesis of periodontal disease. Journal of Clinica Research 26,230-242

Page, RC Schroeder, HE (1976). Phatogenesis of inflammatory periodontal disease. A summaary of current work. Laboratory Investigation 33,235-249

Rodan, GA, Martin, TJ, (1981) Role of osteoblasts in hormonal control of bone resoption: a hypothesis. Calcif Tissue Int 33,349-351

Sakellari, D., Menti, S., & Konstantinidis, A. (2008). Free soluble receptor activator of nuclear factor- kappab ligand in gingival crevicular fluid correlates with distinct pathogens in periodontitis patients. Journal of Clinical Periodontology, 35 (11), 938-943.

Simonet, WS, Lacey, DL, Dunstan, CR, Kelley, M., Chang, MS, Luthy, R. et al. (1997) Osteoprogesterin:

a novel secreted protein involved in the regulation of bone density. Cell 89,309-319

Spolidorio, LC, Spolidorio, DM, Holzhausen, M. (2004) Effects of long-term cyclosporin therapy on the periodontium of rats. Journal or Periodontal Research. 2004 (4), 257-62.

Taubman, MA, Valverde, P., Han, X., Kawai, T. (2005) Immune response: the key to bone resoption in periodontal risease. Journal Periodontology 76, 20033-2041

Teitelbaum, SL, & Ross, FP (2003). Genetic regulation of osteoclast development and function. Nature Reviews.Genetics, 4 (8), 638-649 .

Teng, YT, Nguyen, H., Gao, X., Kong, YY, Gorczynski, RM, Singh, B., Ellen, RP & Penninger, JM (2000) Functional human Tcell immunity and osteoprotegerin ligand alveolar control bone destruction in periodontal infection The Journal of Clinical Investigation 106, R59-R67.

Udagawa, N., Takahashi, N., Akatsu, T., Tanaka, H., Sasaki, T., Nishihara, T., et al. (1990). Origin of osteoclasts: Mature monocytes and macrophages are capable of differentiating into osteoclasts under a suitable microenvironment prepared by bone marrow-derived stromal cells. Proceedings of the National Academy of Sciences of the United States of America, 87 (18), 7260-7264.

Udagawa, N., Takahashi, N., Yasuda, H., Mizuno, A., Itoh, K., Ueno, Y., et al. (2000). Osteoprotegerin produced by osteoblasts is an important regulator in osteoclast development and function. Endocrinology, 141 (9), 3478-3484.

Uito, VJ. (2004) Gingival crevicular fluid: Introduction. Periodontology 2000 6, 9-11

Vernal, R., Chaparro, A., Graumann, R., Puente, J., Valenzuela, MA & Gamonal, J. (2004) Levels of cytokine receptor activator of nuclear factor kB ligand in gingival crevicular fluid in untreated chronic periodontitis patients . Journal of Periodontology

75: 1586-1591.

Vernal, R., Dutzan, N., Hernández, M., Chandía, S., Puente, J., León, R., García, L., Del

Valle, I., Silva, A., Gamonal, J. (2006) High expression levels of receptor activator of nuclear factor-kappa B ligand associated with human chronic periodontitis are mainly secreted by CD4 + T lymphocytes. Journal of Periodontology. 77 (10): 1772-80.

Wada, N., Maeda, H., Tanabe, K., Tsuda, E., Yano, K., Nakamuta, H., et al. (2001). Periodontal ligament cells secrete the factor that inhibits osteoclastic differentiation and function: The factor is osteoprotegerin / osteoclastogenesis inhibitory factor. Journal of Periodontal Research, 36 (1), 56-63.

Walker, DG (1993). Bone resorption restored in osteopetrotic mice by transplants of normal bone marrow and spleen cells. 1975. Clinical Orthopedics and Related Research, (294) (294), 4-6.

Wara-aswapati, N., Surarit, R., Chayasadom, A., Boch, JA, & Pitiphat, W. (2007). RANKL upregulation associated with periodontitis and porphyromonas gingivalis. Journal of Periodontology, 78 (6), 1062- 1069.

Wong, BR, Rho, J., Arron, J., Robinson, E., Orlinick, J., Chao, M., et al. (1997). TRANCE is a novel ligand of the tumor necrosis factor receptor family that activates c-jun N-terminal kinase in T cells. The Journal of Biological Chemistry, 272 (40), 25190-25194.

Yasuda, H., Shima, N., Nakagawa, N., Mochizuki, SI, Yano, K., Fujise, N., Sato, Y.

Goto, M., Yamaguchi, K., Kuriyama, M., Kanno, T., Murakami, A., Tsuda, E., Morinaga, T. & Higashio,

K. (1998b) Identity of osteoclastogenesis inhibitory factor (OCIF) and osteoprotegerin (OPG): a mechanism by which OPG / OCIF inhibits osteoclastogenesis in vitro. Endocrinology 139, 1329-1337.

Yasuda, H., Shima, N., Nakagawa, N., Yamaguchi, K., Kinosaki, M., Mochizuki, S., Tomoyasu, A., Yano, K., Goto, M., Murakami, A., Tsuda, E., Morinaga, T., Higashio, K., Udagawa, N., Takahashi, N. & Suda,

T. (1998a) Osteoclast differentiation factor is a

ligand for osteoprotegerin / osteoclastogenesis inhibitory factor and is identical to TRANCE / RANKL. Proceedings of the National Academy of Sciences of the United States of America 95, 3597-3602
