# Supplementary material for: Impact of periodontal treatment on the RANKL/OPG ratio in crevicular fluid
Source: PLoS One. 2020 Jan 27;15(1):e0227757. doi: 10.1371/journal.pone.0227757 (PMC6984681; doi:10.1371/journal.pone.0227757)
Supplement: S2 Protocol — (DOCX) [file pone.0227757.s002.docx]

**PROYECTO DE TESIS: IMPACTO DEL TRATAMIENTO PERIODONTAL EN EL RATIO RANKL/OPG**


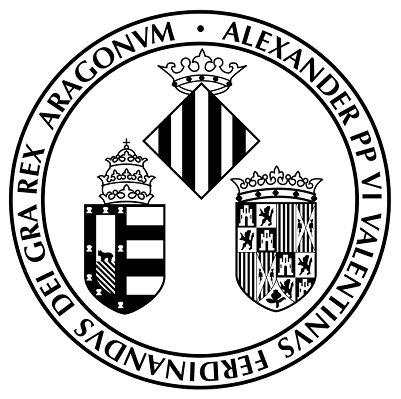


Autor: Ldo. Andrés López Roldán

Tutor y Director: Dr. Francisco Alpiste Illueca.

Unidad docente de Periodoncia. Departamento de Estomatología.

Facultad de Medicina y Odontología. UNIVERSIDAD DE VALENCIA.

**ÍNDICE:**

1. Introducción Pag. 3
2. Justificación e hipótesis de trabajo Pag. 5

1. Objetivos Pag. 7
2. Material y método Pag. 8
3. Bibliografía Pag 14

**1. INTRODUCCIÓN**

Las enfermedades periodontales son un grupo de cuadros clínicos que afectan a las estructuras de soporte del diente, los dos grandes grupos clínicos son la gingivitis y la periodontitis. La gingivitis es una inflamación localizada a nivel de los tejidos del periodonto marginal, sin embargo la periodontitis es un proceso inflamatorio que se extiende a los tejidos de soporte del diente y se caracteriza por la migración apical del epitelio de unión, la destrucción progresiva del ligamento periodontal y del hueso alveolar.

La importancia del estudio y tratamiento de esta enfermedad radica en la alta prevalencia y las consecuencias clínicas; la continua pérdida de soporte periodontal acaba con la pérdida dentaria, pero los efectos de esta patología no sólo quedan circunscritos al campo odontoestomatológico.

En las últimas décadas, han habido grandes avances en el campo de la etiopatogenia de la enfermedad periodontal, siendo esta una entidad compleja donde intervienen varios factores como la microbiología, hábitos higiénicos, genética o factores modificantes sistémicos.

La investigación básica en periodoncia sigue un camino acorde a los avances que se producen en otros campos de la ciencia como en la medicina, biología, bioquímica...etc, de manera que en la disciplina periodontal se ha pasado en pocos años de estudios basados en la anatomopatología a hoy en día, donde el estudio de la patología se aborda cada vez más a nivel genético y molecular.

La principal característica diferenciadora de la periodontitis con respecto al resto de patologías periodontales es la pérdida de inserción periodontal lo que conlleva de forma explícita la resorción de hueso alveolar. En consecuencia, uno de los principales objetivos del tratamiento es evitar que la pérdida de inserción continúe; incluso el objetivo más ambicioso es recuperar el soporte periodontal perdido mediante técnicas de regeneración periodontal.

En cuanto al mecanismo de resorción ósea que ocurre en la periodontitis se sabe que están implicados diversos factores asociados a la respuesta inmunitaria del hospedador así como innumerables señales paracrinas capaces de estimular o inhibir la pérdida ósea.

Pero, sin duda, un gran paso en el conocimiento del metabolismo óseo fue el descubrimiento, a finales de los años 90, del eje RANK/ RANKL/ OPG, siendo éste la piedra angular del proceso de osteoclastogénesis y, por tanto, el regulador de la resorción ósea.

El RANKL (Receptor Activator for Nuclear Factor κ B Ligand**)** es una proteína estimuladora de la diferenciación de los osteoclastos que ejerce su acción mediante la unión al receptor transmembrana RANK (Receptor Activator for Nuclear Factor κ B), presente en los preosteoclastos, en cambio la OPG (osteoprotegerina) actúa como ligando señuelo uniéndose al RANKL e inhibiendo su acción osteoclastogénica.

Este descubrimiento ha supuesto un gran avance en el campo de la medicina, con aplicaciones en múltiples enfermedades que cursan con alteraciones del metabolismo óseo, incluido el campo oncológico.

En el terreno concreto de la periodoncia, las primeras investigaciones surgieron a principio del año 2000, los resultados de estos estudios han demostrado la existencia de valores elevados de RANKL y niveles bajos de OPG a nivel local en los sitios afectados de periodontitis.

De todos los métodos utilizados para la determinación de estas moléculas los estudios con mayor aplicación clínica futura son aquellos que valoran la concentración de estas moléculas a nivel del fluido crevicular por ser una técnica no invasiva.

En la actualidad existen pocas publicaciones que determinen estos mediadores a nivel del fluido crevicular, además se han utilizado distintas metodologías, lo que dificulta la obtención de conclusiones de aplicación clínica. Aunque estas publicaciones han aportado conocimientos importantes para entender mejor los mecanismos de regulación de la remodelación ósea del periodonto, no hemos encontrado ningún estudio que valore el impacto de la terapia periodontal en estos marcadores. Por lo tanto, la intención de este trabajo de investigación es desarrollar un protocolo clínico para la toma de muestras y determinación del RANKL y OPG a nivel del fluido crevicular en pacientes con periodontitis, y posteriormente determinar los cambios que se producen en sus concentraciones cuando se realiza la terapia periodontal básica.

Estudios en esta dirección pueden aportar nueva información de interés clínico que permitan el uso del RANKL y OPG como posibles marcadores de la actividad destructiva de la periodontitis, lo que permitiría utilizarse en el diagnóstico, pronóstico y en el control del tratamiento periodontal.

**2. JUSTIFICACIÓN E HIPÓTESIS DE TRABAJO**

Desde el punto de vista experimental queda clara la importancia del RANKL, RANK y OPG en la regulación de la osteoclastogénesis, lo que ha permitido un mayor entendimiento del metabolismo óseo tanto en condiciones fisiológicas como patológicas.

En el campo de la medicina, el estudio de estas moléculas ha permitido obtener avances importantes tanto en el diagnóstico como en el tratamiento de diversas patologías que cursan con alteraciones del metabolismo óseo. Su interés clínico va desde el campo de la reumatología hasta el de la oncología.

El estudio de posibles determinantes de la actividad destructiva periodontal ha sido uno de los grandes temas de interés en el campo de la periodoncia. Existe gran cantidad de estudios que relacionan los niveles de determinadas citoquinas y de otros mediadores moleculares con la actividad destructiva, pero sin duda el descubrimiento del eje RANKL/RANK/OPG ha permitido entender como se integran las acciones de diversas moléculas implicados en la remodelación ósea, porque es el paso final determinante de la diferenciación y activación ostoclástica.

Las investigaciones publicadas hasta el momento parecen indicar que las concentraciones de RANKL son elevadas en los tejidos periodontales así como en el fluido crevicular de los pacientes con periodontitis. Sin embargo, tenemos poca información acerca de si la concentración de RANKL puede estar relacionada con el grado de severidad de la patología o con las fases de actividad, o si pudiera existir un perfil diferente en las formas clínicas de periodontitis, agresivas o crónicas.

En cuanto a las concentraciones de OPG, parece claro que los niveles de OPG son mayores en aquellos individuos periodontalmente sanos o que sólo padezcan gingivitis. En pacientes con periodontitis los niveles de OPG son menores o incluso marcadamente menor en los casos de periodontitis agresiva.

A la vista de los resultados de las investigaciones publicadas hasta el momento, se puede concluir que el ratio RANKL/OPG es más elevado en periodontitis que en gingivitis o en situación de salud. Según los conocimientos actuales, la células encargadas de la expresión del RANKL a nivel del periodonto son los fibroblastos (gingivales y del ligamento periodontal), los osteoclastos y los osteoblastos; pero en presencia de periodontitis los principales encargados de la expresión del RANKL parecen ser los lingotitos T y B activados. Este hecho explicaría cómo el propio sistema defensivo del hospedador frente a los microorganismos del biofilm bacteriano sería el mecanismo mediador de la osteoclastogénesis (Osteoinmunología).

A pesar de la documentada implicación del RANKL y de la OPG en la enfermedad periodontal aún quedan diversos interrogantes respecto al comportamiento de estos mediadores en la etiopatogenia de esta patología, asi como, a la utilidad clínica que pudieran tener como marcadores de enfermedad periodontal.

Con respecto a esta última cuestión, no se ha estudiado la respuesta que se produce en dichos mediadores moleculares cuando se realiza la terapia periodontal básica.

Nuestra hipótesis principal de trabajo contempla que tratamiento periodontal básico ( raspado y alisado radicular más instrucciones en higiene oral) tiene efecto sobre la concentración de RANKL y OPG en el líquido crevicular de pacientes con patología periodontal, y se produce una disminución en el ratio RANKL/OPG, reduciendo los valores absolutos de RANKL y aumentando los de OPG.

**3. OBJETIVOS**

El objetivo general de este trabajo de investigación es estudiar el impacto del tratamiento periodontal básico sobre el ratio de RANKL/OPG en el fluido crevicular de pacientes con periodontitis.

Los objetivos principales son los siguientes:

1º En el grupo de pacientes con enfermedad periodontal pretendemos estudiar si el grado de salud periodontal esta relacionado con la concentración de RANKL y OPG en el fluido crevicular. Para ello planteamos tres objetivos concretos:

- 1. Determinar las diferencias en los niveles de RANKL y OPG entre localizaciones con y sin inflamación (sangrado al sondaje).
  2. Valorar las diferencias entre localizaciones sanas, con gingivitis y con periodontitis.
  3. Evaluar si el grado de severidad de la periodontitis (inicial, moderada o avanzada) está relacionado con los niveles de RANKL y OPG.
  4. Estudiar el comportamiento de los niveles de estos mediadores moleculares después del tratamiento periodontal básico.

2º El segundo objetivo principal pretende estudiar las diferencias en los niveles de RANKL y OPG en el fluido crevicular entre un grupo de individuos periodontalmente sanos y otro grupo de pacientes con enfermedad periodontal. Para ello planteamos tres objetivos concretos:

- 1. Comparar los niveles medios de RANKL y OPG obtenidos en individuos sanos con los obtenidos en pacientes periodontales.
  2. Comparar los niveles de RANKL y OPG en localizaciones sanas de individuos sanos con los niveles obtenidos en localizaciones sanas de enfermos periodontales.
  3. Contrastar los niveles de estos mediadores moleculares obtenidos en individuos periodontalmente sanos con los pacientes periodontales que han sido tratados con tratamiento periodontal básico.

#### 5. MATERIAL Y MÉTODO

**MUESTRA**

Se estudiará una muestra mínima de 30 individuos, que serán obtenidos del personal de la Facultad y de los pacientes que acuden a primeras visitas en la Unidad de Periodoncia de la Clínica Odontológica de la Universidad de Valencia.

Los 60 individuos serán divididos en dos grupos de estudio :

- - - **Muestra control**: 15 individuos periodontalmente sanos
    - **Muestra de estudio**: 15 pacientes con patología periodontal.

Criterios de inclusión:

Para ser incluidos en el estudio los individuos deben leer y firmar el documento de consentimiento, y se han de comprometer a cumplir fielmente el protocolo del estudio.

**1.- Muestra control:**

Se considerarán individuos sanos aquellos que tanto clínica como radiograficamente no presenten signos ni síntomas de enfermedad periodontal en ninguno de sus dientes.

**2.- Muestra estudio:**

Para ser incluido en la muestra, cada paciente debe haber sido diagnosticado de periodontitis crónica, y además contar al menos con un diente uniradicular por cada una de las siguientes situaciones clínicas:

- *Diente sano*: Profundidades de sondaje menor o igual a 3 mm., sin recesión y sin sangrado al sondaje.
- *Diente con gingivitis*: Profundidades de sondaje igual o menor a 3 mm., sin recesión y con sangrado al sondaje.
- *Diente con periodontitis moderada*: Pérdida de inserción mayor de 3 mm. y menor a 6 mm.
- *Diente con periodontisis severa*: Pérdida de inserción mayor de 6 mm.

Criterios de exclusión:

1.- Pacientes con periodontitis agresiva.

2.-Presencia de alteraciones sistémicas o toma de fármacos que alteren el metabolismo óseo (osteoporosis, artritis, tratamiento hormonal, bifosfonatos, antiiflamatorios, inmunosupresores...etc).

3.-Haber recibido tratamiento antibiótico, antiinflamatorio, anticonceptivo en los tres últimos meses.

4.- Trauma oclusal primario o secundario en alguno de los dientes incluidos en los estudios.

5.- Haber recibido tratamiento periodontal en los últimos 3 meses.

6.- Estar en tratamiento ortodóncico.

Criterios de exclusión durante el estudio:

1.- Toma de cualquier fármaco que pueda alterar la respuesta al tratamiento periodontal (antiinflamatorios, antibióticos, colutorios antisépticos).

2.- Cualquier fármaco que pueda alterar el metabolismo óseo.

De la misma manera será criterio de exclusión la imposibilidad por parte del individuo del cumplimiento estricto de las normas del protocolo.

**MATERIAL :**

- Sonda periodontal (Williams 10mm. Hu-Friedry ®)
- Sistema de paralelización radiográfica O-Ring (Densply®)
- Aparato de radiografias intraoral (E-wood®, Haigol-Dong, Korea)
- Perio paper strips ®: tiras de papel especiales para recoger volúmenes de fluido crevicular de 0-1,2 ml (Oraflow®, New York, USA)
- Periotron 8000®: micro-medidor de humedad electrónico especialmente diseñado para valorar el fluido crevicular, el fluido de la bolsa periodontal y el fluido salival (Oraflow®, New York, USA)
- Periotron professional: Sofware diseñado para pasar los valores dados por el Periotron 8000® a mililitros (Oraflow®, New York, USA)
- Viales de reacción 1,5 ml (Ependorff®)
- Pipetas de precisión calibradas de 10-1000 μl y puntas desechables (Ependorff®)
- Agua destilada o desionizada
- PBS ( Fosfato buffer salino)
- Inhibidores de las proteasas ( C.N. P8340 Sigma, Missouri, USA)
- Kit Elisa sRANKL y OPG ( Biomedica Medizinprodukte®, Austria)
- Lavador automático de placas
- Lector de ELISA con capacidad para leer absorbancias desde 450 nm a 620 nm.

**PARÁMETROS CLÍNICOS:**

- **Datos de la historia clínica:**
  - - Edad del paciente
    - Patologías sistémicas
    - Medicación
    - Habito tabáquico
    - Hábitos higiénicos ( material, frecuencia, técnica y tiempo)
- **Exploración periodontal:**
  - - Profundidad de sondaje: con una sonda periodontal manual Williams milimetrada se registraran seis puntos por diente (disto-vestibular, medio-vestibular, mesio-vestibular, disto-palatino, medio-palatino y mesio- palatino).
    - Recesión: se medirá la distancia desde la línea amelocementaria al margen gingival y se registrarán en los mismos puntos citados anteriormente (disto-vestibular, medio-vestibular, mesio-vestibular, disto-palatino, medio-palatino y mesio- palatino).
    - Nivel de inserción clínica: Sumando los parámetros anteriormente citados podemos hallar este parámetro por cada punto.
    - Índice dicotómico de sangrado al sondaje(Índice de Hemorragia).
    - Índice de movilidad de Miller.
    - Índice de placa de Silness y Löe.
- **Exploración radiográfica:**
  - - El en grupo casos, se realiza una serie radiográfica completa formada por 18 radiografías de las cuales 14 son periapicales de incisivos, caninos, premolares y molares de todos los cuadrantes y 4 aletas de mordida verticales de premolares y molares. Todas ellas se realizan con técnica radiográfica paralelizada mediante la utilización del sistema de paralelización O-Ring y aparato de rayos X intraoral.
    - En el grupo control sólo se realizarán dos aletas de mordidas horizontales para confirmar el diagnóstico clínico de ausencia de enfermedad periodontal

**TOMA DE MUESTRAS DEL FLUIDO CREVICULAR Y PROCESADO:**

El mismo día de la exploración clínica se recogen 4 muestras de fluido crevicular de cada uno de los dientes uniradiculares con las 4 situaciones clínicas descritas en los criterios de inclusión.

- - - Eliminar la placa supragingival con una cureta estéril sin tocar la encia, lavar con abundante agua, aislar con rodillos de algodón y secar con aire para evitar la contaminación con saliva.
    - Se inserta la punta de Perio Paper® hasta notar resistencia y dejarla durante 30 segundos. NOTA: Si el papel se mancha de sangre o de detritos la muestra esta contaminada y hay que volver a tomarla.
    - Medir mediante el Periotron 8000® y el sofware adecuado el volumen de cada una de las muestras.
    - Colocar las puntas de papel en un tubo Eppendorf esterilizado, añadir a la muestra 100 microlitros de buffer ( PBS junto a inhibidores de las proteasas) y se centrifugar a 15,000 durante 5 min.
    - Añadir otros 100 microlitros de buffer y volver a centrifugar a 15,000 durante 5 min.
    - Conservar los 200 microlitros de cada muestra a – 80ºC hasta el momento de realizar el test ELISA.

## PRUEBAS DE ENSAYO ELISA

Determinación de la concentración de RANKL y OPG en las muestras se realizará mediantes técnicas de ensayo por inmunoabsorción ligado a enzimas ( Enzyme-Linked ImmunoSorbent Assay), concretamente se van ha utilizar kits preparados de la casa comercial Biomedica Medizinprodukte®. Los análisis se realizarán siguiendo las recomendaciones del fabricante y la concentración se determinara una vez realizada la cuantificación del RANKL y la OPG junto al volumen hallado con el periotron.

**INTERVENCIÓN EN LA MUESTRA CASOS:**

En el grupo de pacientes pertenecientes a la muestra casos, después de haber recogido todos los datos clínicos, radiológicos y muestras de fluido crevicular, se le realizará tratamiento periodontal básico. El tratamiento consta de raspado y alisado radicular de los 4 cuadrantes sin la utilización antisépticos o antibióticos de acción local. Como parte del tratamiento, a los pacientes se les instruirá en higiene oral, tanto en técnicas de cepillado como en el uso de medidas de higiene interproximal. A las 4 semanas se les citará para revaluar clínicamente y tomar nuevamente muestras del líquido crevicular.

**SECUENCIA DEL PROTOCOLO:**

Día 0 .- Comprobación de adecuación a los criterios de inclusión y de exclusión del estudio. Al paciente se le explicará en que consiste el estudio, el protocolo a seguir y el consentimiento del paciente. Realización de anamnesis, y recogida de datos clínicos y radiológicos.

Día 7.- Se tomarán las muestras de fluido crevicular, de las zonas seleccionadas y se realizará el procesado de las muestras.

Día 14.- Tratamiento periodontal (grupo casos): Raspado y alisado radicular de 2 cuadrantes e instrucciones en higiene oral.

Día 21 .- Tratamiento periodontal (grupo casos) : Raspado y alisado radicular de los 2 cuadrantes restantes e instrucciones en higiene oral.

Día 49.- Grupo casos: Revaluación periodontal, registros clínicos y toma de muestras de fluido crevicular. Procesado de las muestras

7. BIBLIOGRAFÍA

Abu-Amer, Y., Erdmann, J., Alexopoulou, L., Kollias, G., Ross, F.P., Teitelbaum, S.L. (2000) Tumor necrosis factor receptors types 1 and 2 differentially regulate osteoclastogenesis. Journal of Biological Chemistry.275(35),27307-10

Anderson, D.M., Maraskovky, E., Billingsley W.L., Dougall W.C., Tometsko M.E., Rous E.R., et al. ( 1997) A homologue of the TNF receptor and its ligand enhance T-cell growth and dendritic-cell function. Nature 390, 175-179

Arron, J.R., Choi, Y.(2000) Bone versus immune system. Nature 30 ;408(6812),535-6

Armitage, G.C.(1999) Development of a classification system for periodontal diseases

and conditions. Annals of Periodontolgy 4,(1):1-6.

Bar-Shavit, Z. (2008). Taking a toll on the bones: Regulation of bone metabolism by innate immune regulators. Autoimmunity, 41(3), 195-203.

Bostanci, N., Ilgenli, T., Emingil, G., Afacan, B., Han, B., Toz, H., Atilla, G., Hughes, F. J. & Belibasakis, G. N. (2007a) Gingival crevicular fluid levels of RANKL& OPG in periodontal diseases: implications of their relative ratio. Journal of Clinical Periodontology 34, 370–376.

Bostanci, N., Ilgenli, T., Emingil, G., Afacan, B., Han, B., To¨z, H., Berdeli, A., Atilla, G., McKay, I., Hughes, F. & Belibasakis, G. (2007b) Differential expression of RANKL

and OPG mRNA in periodontal diseases. Journal of Periodontal Research. 42, 287-293

Boyce, B.F., Xing L. (2007) Biology of RANK, RANKL, and osteoprogesterin. Arthritis Research & Therapy 9, 1-7

Brecx, M.C., Fröhlicher, I., Gehr, P., Lang, NP.(1988) Stereological observations on

long-term experimental gingivitis in man. Journal Of Clinical Periodontoly.15(10), 621-7.

Choi, Y., Woo, K.M., Ko, S.H., et al.(2001) Osteoclastogenesis in enhanced by activated B cells but suppressed by activated CD8+ T cells. European Journal Inmunology. 31, 2179-2188

Crotti, T., Smith, M. D., Hirsch, R., Soukoulis, S., Weedon, H., Capone, M., Ahern, M. J. & Haynes, D. (2003) Receptor activator NF kappaB ligand (RANKL) and osteoprotegerin (OPG) protein expression in periodontitis. Journal of Periodontal Research 38, 380–387.

Dougall, W.C., Glaccum, M., Charrier, K., Rohrbach, K., Brasel, K., et all.(1999) RANK is essential for osteoclast and lymph node development. Genes and Development.13(18),2412-24

Ferrer J., Tovar, I., Martínez, P. (2002) Osteoprotegerina y Sistema RANKL/RANK: ¿el futuro del metabolismo óseo? Anales de Medicina Interna 19, 385-388.

Fuller, K., Murphy, C., Kirstein, B., Fox, S.W., Chambers, T.J.(2002) TNFalpha potently

activates osteoclasts, through a direct action independent of and strongly

synergistic with RANKL. Endocrinology 143(3),1108-18

Garlet, G. P., Cardoso, C. R., Silva, T. A., Ferreira, B. R., Avila-Campos, M. J., Cunha, F. Q., et al. (2006). Cytokine pattern determines the progression of experimental periodontal disease induced by actinobacillus actinomycetemcomitans through the modulation of MMPs, RANKL, and their physiological inhibitors. Oral Microbiology and Immunology, 21(1), 12-20.

Glossary of Periodontal Terms. 4^th^ Edition. The American Academy of Periodontology 2001

Grant, P. R., & Mulvihill, J. E. (1972). The fine structure of gingivitis in the beagle. 3. plasma cell infiltration of the subepithelial connective tissue. Journal of Periodontal Research, 7(2), 161-172.

Griffiths, G. (2004) Formación , acumulación e importancia del líquido crevicular gingival. Periodontology 2000 6 32-41

Hasegawa, T., Yoshimura, Y., Kikuiri, T., Yawaka, Y., Takeyama, S., Matsumoto, A., et al. (2002). Expression of receptor activator of NF-kappa B ligand and osteoprotegerin in culture of human periodontal ligament cells. Journal of Periodontal Research, 37(6), 405-411.

Hodge, P., & Michalowicz, B. (2001). Genetic predisposition to periodontitis in children and young adults. Periodontology 2000, 26, 113-134.

Horton, J. E., Raisz, L. G., Simmons, H. A., Oppenheim, J. J., & Mergenhagen, S. E. (1972). Bone resorbing activity in supernatant fluid from cultured human peripheral blood leukocytes. Science (New York, N.Y.), 177(51), 793-795.

Kanzaki, H., Chiba, M., Shimizu, Y., & Mitani, H. (2001). Dual regulation of osteoclast differentiation by periodontal ligament cells through RANKL stimulation and OPG inhibition. *Journal of Dental Research, 80*(3), 887-891.

Kawai, T., Matsuyama, T., Hosokawa, Y., Makihira, S., Seki, M., Karimbux, N. Y., et al. (2006). B and T lymphocytes are the primary sources of RANKL in the bone resorptive lesion of periodontal disease. The American Journal of Pathology, 169(3), 987-998.

Khosla, S. (2001) Minireview: The OPG/RANKL/RANK system. Endocrinology 142, 5050–5055

Kinane, DF. Lindhe, J. (2005) Clasificación de las enfermedades periodontales. En: Lindhe, J. Periodoncia clínica e implantologia odontológica. Ed. Panamericana, Buenos Aires. P. XII

Kinane, DF., Adonogianaki, E., Moughal, N., Winstanley, FP., Mooney, J., Thornhill, M. (1991). Immunocytochemical characterization of cellular infiltrate, related endothelial changes and determination of GCF acute-phase proteins during human experimental gingivitis. Journal of Periodontal Research. 26(3), 286-8

Kitaura, H., Sands, M.S., Aya, K., Zhou, P., Hirayama, T., Uthgenannt, B., Wei, S., Takeshita, S., Novack, D.V., Silva, M.J., Abu-Amer, Y., Ross, F.P., Teitelbaum, S.L.(2004) Marrow stromal cells and osteoclast precursors differentially contribute to TNF-alpha-induced osteoclastogenesis in vivo. Journal of Immunology.173(8),4838-46

Kong, Y.Y., Boyle, W.J., Penninger, J.M (2000). Osteoprotegerin ligand: a regulator of immune responses and bone physiology. Immunology Today. 10, 495-502

Lam, J., Takeshita, S., Barker, J.E., Kanagawa, O., Ross, F.P., Teitelbaum, S.L.(2000) TNF-alpha induces osteoclastogenesis by direct stimulation of macrophages exposed to permissive levels of RANK ligand. Journal of Clinical Investigation. 106(12),1481-8

Lacey, D. L., Timms, E., Tan, H. L., Kelley, M. J., Dunstan, C. R., Burgess, T., et al. (1998). Osteoprotegerin ligand is a cytokine that regulates osteoclast differentiation and activation. Cell, 93(2), 165-176.

Lee, S. H., Kim, T. S., Choi, Y., & Lorenzo, J. (2008). Osteoimmunology: Cytokines and the skeletal system. BMB Reports, 41(7), 495-510.

Lerner, U.H.,(2006)Inflammation-induced bone remodeling in periodontal disease and the influence of post-menopausal osteoporosis. Journal of Dental Research. 85(7),596-607.

Li, Y., Toraldo, G., Li, A., Yang, X., Zhang, H., Qian, W. P., et al. (2007). B cells and T cells are critical for the preservation of bone homeostasis and attainment of peak bone mass in vivo. Blood, 109(9), 3839-3848.

Löe, H., Theilade, E., Jesen S.B. (1965). Experimental gingivitis in man. Journal of Periodontology 36, 177-187

Lorenzo, J., Horowitz, M., & Choi, Y. (2008). Osteoimmunology: Interactions of the bone and immune system. Endocrine Reviews, 29(4), 403-440.

Lu, H.-K., Chen, Y.-L., Chang, H.-C., Li, C.-L. & Kuo, M.Y.-P (2006) Identification of the osteoprotegerin/receptor activator of nuclear factor- kappa B ligand system in gingival crevicular fluid and tissue of patients with chronic periodontitis. Journal of Periodontal Research 41: 354–360.

Martínez H, Jiménez F, Estrada C, Anaya J, Quiñones M. Citoquinas y quimioquinas. En: Anaya J, Shoenfeld Y, Correa P. Autoinmunidad y Enfermedad autoinmune. Ed. Corporación para investigaciones biológicas; 2005. p. 121-132

Michalowicz, B. S., Diehl, S. R., Gunsolley, J. C., Sparks, B. S., Brooks, C. N., Koertge, T. E., et al. (2000). Evidence of a substantial genetic basis for risk of adult periodontitis. Journal of Periodontology, 71(11), 1699-1707.

Mogi, M., Otogoto, J., Ota, N. & Togari, A. (2004) Differential expression of RANKL

and osteoprotegerin in gingival cervicular fluid of patients with periodontitis. Journal of Dental Research 83, 166–169.

Myers, D.E., Collier, F.M., Minkin, C., Wang, H., Holloway, W.R., Malakellis, M., Nicholson,G.C.(1999) Expression of functional RANK on mature rat and human osteoclasts. FEBS Letters.463(3),295-300

Nagasawa, T., Kobayashi, H., Kiji, M., Aramaki, M., Mahanonda, R., Kojima, T., Murakami, Y., Saito, M., Morotome, Y., Ishikawa, I. (2002) LPS-stimulated human gingival fibroblasts inhibit the differentiation of monocytes into osteoclasts through the production of osteoprotegerin. Clinical and Experimental Immunology.130(2),338-44

Ogasawara, T., Yoshimine, Y., Kiyoshima, T., Kobayashi, I., Matsuo, K., Akamine, A., et al. (2004). In situ expression of RANKL, RANK, osteoprotegerin and cytokines in osteoclasts of rat periodontal tissue. Journal of Periodontal Research, 39(1), 42-49.

Page, R.C. (1991) The role of inflammatory mediators in the patogénesis of periodontal disease. Journal of Clinica Research 26,230-242

Page, R.C. Schroeder, H.E. (1976). Phatogenesis of inflammatory periodontal disease. A summaary of current work. Laboratory Investigation 33,235-249

Rodan, G.A., Martin, T.J., (1981) Role of osteoblasts in hormonal control of bone resoption:a hypothesis. Calcif Tissue Int 33,349-351

Sakellari, D., Menti, S., & Konstantinidis, A. (2008). Free soluble receptor activator of nuclear factor-kappab ligand in gingival crevicular fluid correlates with distinct pathogens in periodontitis patients. Journal of Clinical Periodontology, 35(11), 938-943.

Simonet, W.S., Lacey, D.L., Dunstan, C.R., Kelley, M., Chang, M.S., Luthy, R. et al. (1997) Osteoprogesterin: a novel secreted protein involved in the regulation of bone density. Cell 89,309-319

Spolidorio, L.C., Spolidorio, D.M., Holzhausen, M.(2004) Effects of long-term cyclosporin therapy on the periodontium of rats. Journal or Periodontal Research. 2004 (4), 257-62.

Taubman, M.A., Valverde, P., Han, X., Kawai, T.(2005) Inmune response:the key to bone resoption in periodontal risease. Journal Periodontology 76, 20033-2041

Teitelbaum, S. L., & Ross, F. P. (2003). Genetic regulation of osteoclast development and function. Nature Reviews.Genetics, 4(8), 638-649.

Teng, Y. T., Nguyen, H., Gao, X., Kong, Y. Y., Gorczynski, R. M., Singh, B., Ellen, R. P. & Penninger, J. M. (2000) Functional human Tcell immunity and osteoprotegerin ligand control alveolar bone destruction in periodontal infection. The Journal of Clinical Investigation 106, R59–R67.

Udagawa, N., Takahashi, N., Akatsu, T., Tanaka, H., Sasaki, T., Nishihara, T., et al. (1990). Origin of osteoclasts: Mature monocytes and macrophages are capable of differentiating into osteoclasts under a suitable microenvironment prepared by bone marrow-derived stromal cells. Proceedings of the National Academy of Sciences of the United States of America, 87(18), 7260-7264.

Udagawa, N., Takahashi, N., Yasuda, H., Mizuno, A., Itoh, K., Ueno, Y., et al. (2000). Osteoprotegerin produced by osteoblasts is an important regulator in osteoclast development and function. Endocrinology, 141(9), 3478-3484.

Uito, VJ. (2004) Líquido crevicular gingival: Introducción. Periodontology 2000 6, 9-11

Vernal, R., Chaparro, A., Graumann, R., Puente, J.,Valenzuela, M.A. & Gamonal, J. (2004) Levels of cytokine receptor activator of nuclear factor kB ligand in gingival crevicular fluid in untreated chronic periodontitis patients. Journal of Periodontology

75: 1586–1591.

Vernal, R., Dutzan, N., Hernández, M., Chandía, S., Puente, J., León, R., García, L., Del

Valle, I., Silva, A., Gamonal, J.(2006) High expression levels of receptor activator of nuclear factor-kappa B ligand associated with human chronic periodontitis are mainly secreted by CD4+ T lymphocytes. Journal of Periodontology. 77(10):1772-80.

Wada, N., Maeda, H., Tanabe, K., Tsuda, E., Yano, K., Nakamuta, H., et al. (2001). Periodontal ligament cells secrete the factor that inhibits osteoclastic differentiation and function: The factor is osteoprotegerin/osteoclastogenesis inhibitory factor. Journal of Periodontal Research, 36(1), 56-63.

Walker, D. G. (1993). Bone resorption restored in osteopetrotic mice by transplants of normal bone marrow and spleen cells. 1975. Clinical Orthopaedics and Related Research, (294)(294), 4-6.

Wara-aswapati, N., Surarit, R., Chayasadom, A., Boch, J. A., & Pitiphat, W. (2007). RANKL upregulation associated with periodontitis and porphyromonas gingivalis. Journal of Periodontology, 78(6), 1062-1069.

Wong, B. R., Rho, J., Arron, J., Robinson, E., Orlinick, J., Chao, M., et al. (1997). TRANCE is a novel ligand of the tumor necrosis factor receptor family that activates c-jun N-terminal kinase in T cells. The Journal of Biological Chemistry, 272(40), 25190-25194.

Yasuda, H., Shima, N., Nakagawa, N., Mochizuki, S. I., Yano, K., Fujise, N., Sato, Y.,

Goto, M., Yamaguchi, K., Kuriyama, M., Kanno, T., Murakami, A., Tsuda, E., Morinaga,T. & Higashio, K. (1998b) Identity of osteoclastogenesis inhibitory factor (OCIF) and osteoprotegerin (OPG): a mechanism by which OPG/OCIF inhibits osteoclastogenesis in vitro. Endocrinology 139, 1329–1337.

Yasuda, H., Shima, N., Nakagawa, N., Yamaguchi, K., Kinosaki, M., Mochizuki, S., Tomoyasu, A., Yano, K., Goto, M., Murakami, A., Tsuda, E., Morinaga, T., Higashio, K., Udagawa, N., Takahashi, N. & Suda, T. (1998a) Osteoclast differentiation factor is a

ligand for osteoprotegerin/osteoclastogenesis inhibitory factor and is identical to TRANCE/RANKL. Proceedings of the National Academy of Sciences of the United States of America 95, 3597–3602
